# Fine Mapping of the *MAP2K5* Region Identified rs7175517 as a Causal Variant Related to BMI in China and the United Kingdom Populations

**DOI:** 10.3389/fgene.2022.838685

**Published:** 2022-03-16

**Authors:** Ce Lu, Hai-Jun Wang, Jie-Yun Song, Shuo Wang, Xue-Ying Li, Tao Huang, Hui Wang

**Affiliations:** ^1^ Department of Epidemiology, School of Public Health, Nanjing Medical University, Nanjing, China; ^2^ Department of Maternal and Child Health, School of Public Health, Peking University, Beijing, China; ^3^ Institute of Child and Adolescent Health, School of Public Health, Peking University, Beijing, China; ^4^ Department of Epidemiology and Biostatistics, School of Public Health, Peking University, Beijing, China

**Keywords:** BMI, eQTL, fine mapping, GWAS, MAP2K5

## Abstract

**Background:** Genome-wide association studies (GWASs) have consistently identified *MAP2K5* as an obesity susceptibility gene. To deepen our understanding of the potential causal genetic variants of this region, a fine-mapping study of *MAP2K5* was conducted.

**Methods** and **Results:** SNPs rs7175517 (G > A) and rs4776970 (T > A) were identified as the leading SNPs associated with BMI in both Chinese and the United Kingdom populations. Second, colocalization of GWAS and expression quantitative trait loci (eQTL) analyses and bioinformatic analyses indicated that rs7175517 is the functionally leading variant in the *MAP2K5* gene region. Dual-luciferase assays indicated that the G allele of rs7175517 reduced the mRNA expression of *MAP2K5* in HEK293T cells. The possible mechanism was that the G allele interacted with more RNA repressors from nuclei extracts, which was evidenced by electrophoretic mobility shift assays (EMSAs). Furthermore, the pathway enrichment analyses of the products from DNA pull-down and protein mass spectrometry demonstrated that the G allele of rs7175517 might interact with RNA catabolic or splicing transcription factors, which consequentially increased adiposity deposition.

**Conclusion:** SNP rs7175517 of the *MAP2K5* gene was the putative causal variant associated with BMI. More precisely designed *in vitro* or animal experiments are warranted to further delineate the function of *MAP2K5* in adipogenesis.

## Introduction

Obesity is a serious health epidemic globally. A recent study of 195 countries estimated that 2.2 billion people were overweight or obese in 2015 (Collaborators et al., 2017). The rapid rise of obesity is also a major public health problem in developing countries, including China. According to the most recent national survey, more than half of Chinese adults are either overweight or obese (SCIO, 2020). The estimated attribution percentage of overweight and obesity-associated noncommunicable disease (NCD) deaths increased from 5.7% in 1990 to 11.1% in 2019 in China (Institute for Health Metrics and Evaluation Global Health Data Exchange, 2021). The latest Chinese national prevalence of overweight and obesity in children and adolescents, based on the Chinese BMI screening criteria, were 6.8 and 3.6% in children under 6 years old and 11.1 and 7.9% in children aged 6–17 years, respectively (Pan et al., 2021).

Although the increase in obesity prevalence was considered to be caused by changes in the external environment, such as a hypercaloric diet and sedentary lifestyle, genetic factors and gene–environment interactions still play a critical role in obesity [4]. The heritability of body mass index (BMI) can reach 40–70%. Several genome-wide association studies (GWASs) have revealed certain susceptible genetic polymorphisms, such as fat-mass and obesity-associated gene (*FTO*) rs1421085, *SH2B* adapter protein 1 (*SH2B1*) rs4788099 and mitogen-activated protein kinase kinase 5 (*MAP2K5*, the encoded protein named MEK5) rs2241423 (Thorleifsson et al., 2009; Speliotes et al., 2010). Since then, a number of studies have pinpointed that *MAP2K5* rs2241423 is associated with both childhood and adulthood obesity in different Asian populations and Caucasian, which most results were consistent (Speliotes et al., 2010; Dorajoo et al., 2012; Rask-Andersen et al., 2012; Wang et al., 2016; Lee et al., 2017). It seems that genetic variants of MAP2K5 played consistent role both in adults and children. In 2015, a MetaboChip meta-analysis for BMI identified 56 more novel loci and confirmed the association between the MAP2 protein complex and obesity (Locke et al., 2015). In 2017, Abadi et al. (2017) selected 37 BMI-associated SNPs to observe the BMI percentile distribution with 75,230 European ancestry participants and revealed that rs997295 of *MAPK5* had a positive association with BMI. Recently, Pan et al. (2018) applied the promoter capture Hi-C in human adipocytes to decipher the transcription-regulation mechanism that contributed to adipogenesis. The results revealed that *MAP2K5* rs4776984 was a *cis*-expression quantitative trait(eQTL)-eGene in the regulation of BMI. One *in vitro* experimental study demonstrated that MEK5(encoded by *MAP2K5*) is the only known activator of *ERK5*, a key regulator of adipogenesis via the protein kinase cAMP-dependent (PKA) signaling axis (Zhu et al., 2014). However, until now, no further study has been carried out to thoroughly delineate the association of variants within the *MAP2K5 gene region* with obesity.

Additionally, like other complex diseases, most of the *MAP2K5* variants reported by GWAS lie within noncoding regions, probably due to linkage disequilibrium (LD), which makes causal variant inference and consequential functional evaluation complicated (Hutchinson et al., 2020). Fine mapping is a complementary method for GWAS that can further elucidate the risk region/gene by investigating as many variants as possible either by imputation or sequencing for more detailed analysis and be combined with functional annotation to illustrate the biological mechanism of variants (Schaid et al., 2018). Therefore, the present study focused on the *MAP2K5* gene region and utilized Chinese children and the United Kingdom Biobank (UKB) population to explore the causal variants among the *MAP2K5* gene regions associated with body mass index (BMI). Furthermore, *in*-*silico* functional annotation, bioinformatic colocalization analyses and *in vitro* experiments were performed to reveal possible molecular mechanisms.

## Materials and Methods

### Study Population

A two‐stage case‐control study was conducted. In the discovery stage, participants were recruited from two independent case–control studies in the urban areas of Beijing, China. First, the study of adolescent lipids, insulin resistance and candidate genes (ALIR) included 151 normal-weight, 400 overweight and 386 obese children aged 7–18 years old. Second, the baseline of the Comprehensive Prevention Project for Overweight and Obese Adolescents (CPOOA) collected 456 normal-weight, 318 overweight and 319 obese children aged 14–17 (Wang et al., 2008; Wang et al., 2010). Individuals with any cardiovascular or metabolic-related diseases were excluded as well. BMI was calculated by dividing weight (kg) by the square of height (m^2^). According to the BMI percentile criteria, children with an age- and sex-specific BMI ≥95th percentile were classified into the obese group, and those with a BMI between the 85th and 95th percentiles were classified into the overweight group, whereas those with a BMI between the 15th and 85th percentiles were normal weight. Children with BMI ≥97th percentile were defined as severely obese (Ji and Working Group on Obesity in China, 2005). Both studies were approved by the Ethics Committee Board of Peking University Health Science Center.

In the replication stage, information was extracted from the UKB database. The UKB database is a cohort of approximately half a million individuals aged 40–69 years across the United Kingdom. The UKB data are available on application to the UKB (www.ukbiobank.ac.uk/). This research was conducted using the UKB data under Application Number 44430. A BMI between 18.5 and 25 is classified as normal, 25–30 as overweight, 30–35 as obese and more than 35 as severely obese (Team, 2005). The study only included Caucasian people and proposed individuals with no blood relationship. The first, second and third principal component (PC) filtering was applied, and those with information on age, sex, BMI and Townsend deprivation index individuals were selected. Finally, 264,838 nonrelated individuals of self-reported British descent from the United Kingdom Biobank were included in the present study. The summary-level GWAS data used in the present study are publicly available. Therefore, no specific ethical approval is needed.

### Genotyping Quality Control and Imputation

In the discovery stage, 9 variants located in *MAP2K5* genes, with 2 variants from published literature (Speliotes et al., 2010; Wen et al., 2012) and 7 Tag SNPs based on the CHB database from the 1,000 Genomes Project, were selected for genotyping. Genotyping was performed with matrix-assisted laser desorption ionization time of flight mass spectrometry (MALDI-TOF MS, Agena, San Diego, CA, United States). The call rates were above 99.4%, and 3 variants were excluded for strong linkage disequilibrium (*r* ^2^ > 0.80) in the present study. The remaining 6 variants are shown for basic genotyping information (Supplementary Table S1).

In the replication stage, the genotype data of SNPs located in the *MAP2K5* region were extracted from the UKB GWAS database. SNP selection was conducted under the following criteria: 1) imputation quality score (INFO) ≥ 0.9; 2) genotyping call rate ≥  95%; 3) minor allele frequency (MAF) in controls ≥0.01; and 4) Hardy–Weinberg equilibrium (HWE) ≥1 × 10^−6^. Finally, 2,994 SNPs were included in the subsequent analyses. The quality control was implemented with Plink (v1.90).GWAS data of the UKB were measured with Applied Biosystems™ United Kingdom BiLEVE Axiom™ Array (49950 participants) or Applied Biosystems™ United Kingdom Biobank Axiom Array (438,427 participants) by Affymetrix, which could acquire more than 800,000 markers (Bycroft et al., 2018). Imputation was performed with SHAPEIT3 and IMPUTE3 based on merged panels of UK10K and 1,000 Genomes phase3 (Howie et al., 2009; Delaneau et al., 2013).

### eQTL Analyses and *In-Silico* Functional Annotations

eQTL associations were identified by searching the Genotype‐Tissue Expression Project (GTEx; http://www.gtexportal.org/home/, database V8 release) (Consortium, 2013). Variant‐gene paired eQTL analysis results were conducted in subcutaneous adipose and whole blood tissues.

To explore the potential molecular functions of the colocalized genes and corresponding variants, we performed *in-silico* functional annotations with several prediction aspects, including histone modification sites (H3K4me1, H3K4me3 and H3K27ac) from the Encyclopedia of DNA Elements (ENCODE). All outcomes were visualized in UCSC browser (Haeussler et al., 2019; Hallikas et al., 2006). Finally, pathway enrichment analyses were adopted to explore which signaling pathway the transcription factor was involved via GO, KEGG and REAC pathways (Reimand et al., 2019).

### Dual Luciferase Reporter Assays (DLRA)

Luciferase constructs encompassed surrounding sequences of rs7175517(G/A) (NCBI: chr15:68077130-68078130,GRCh37) was cloned into the pGL3-Promoter vector (Promega, Madison, WI, United States). The luciferase constructs were synthesized by the Youbio Biological technology Co. Ltd. (Changsha, China).The constructed plasmids were sequenced to confirm the accuracy (GenScript Biotechnology Co. Nanjing, China). HEK293T cells were plated into 24-well plates in each well (7.5 × 10^4^) and cotransfected with the plasmids (100 ng/well) of interest the next day with pRL-SV40 Renilla Luciferase Control Vector (10 ng/well, Promega, Madison, WI, United States) using Lipofectamine 2000 reagent (Invitrogen, Carlsbad, CA, United States). After 48 h of culture, the cells were lysed, and luciferase activity was measured using the Dual-Luciferase Reporter Assay System (Promega, Madison, WI, United States). Relative luminescent signals were calculated by normalizing luciferase signals with Renilla signals. In total, 3 independent transfection experiments with triplicates for each condition were conducted.

### Electrophoretic Mobility Shift Assay (EMSA)

Nuclear extracts were prepared from HEK293T cells using the NE-PER Nuclear and Cytoplasmic Extraction kit (Thermo Scientific, Waltham, MA, United States). DNA oligonucleotides for each variant were synthesized with 5′-biotin labeling and HPLC purified by Genscript Biotechnology Co. (Nanjing, China; probe sequences are listed in Supplementary Table S5). Double-stranded DNA probes were prepared by combining sense and antisense oligonucleotides, heat annealing, and slow cooling. Probes and HEK293T cell nuclear extracts were then incubated by using the LightShift EMSA Optimization & Control Kit (Thermo Scientific) at 4°C for 20 min. For competition assays, unlabeled competitors at 2-fold, 5-fold or 100-fold excess oligonucleotides were added to the reaction mixture 10 min before the addition of labeled probes. After incubation, binding reactions were separated on a 6% polyacrylamide gel, transferred blots were developed using the Chemiluminescent Nucleic Acid Detection Module (Thermo Scientific), and signals were visualized with the ChemiDoc XRS + scanner (BIO-RAD, Louisville, KY, United States).

### DNA Pull-Down and Protein Mass Spectrometry

The biotin-labeled probe and magnetic beads were placed in a 4°C freezer and incubated for 6–8 h. The nuclear extracts were incubated with the magnetic bead DNA probe complexes placed in a 4°C freezer and incubated overnight after washing to remove nonspecifically bound proteins. Finally, the eluate was subjected to elution to obtain the product of interest, which was then subjected to protein mass spectrometry to identify the protein. Protein mass spectrometry was conducted in the central lab of Nanjing Medical University.

### Statistical Analyses

Hardy-Weinberg equilibrium of all the genotypes was analyzed with the χ^2^ test. Logistic regression and linear regression analyses were conducted to analyze the effect of genetic variants on overweight and obesity (categorical variables) or BMI, individually. The adjusted covariables were age, sex and study population. For the UKB data analysis, age, sex, income, educational attainment (income and educational attainment were replaced by the Townsend deprivation index for the forward stepwise regression) and the top three principal components were adjusted for logistic and linear regressions. All genetic regression analyses were performed under an additive model and conducted with Plink software (v.1.9). The linkage disequilibrium (LD) between variants was tested by calculating *r* ^2^ with Haploview 4.2. In terms of genetic association analyses, statistical significance was considered when *p* values < 5 × 10^−8^. For the *in vitro* experiments, an unpaired Student’s *t* test was used to compare the mean value of different conditional triplicates, and two-sided *p* values < 0.05 were considered significant unless otherwise specified. Then, functional enrichment analysis was performed using g:Profiler (version e104_eg51_p15_3922dba) with g:SCS multiple testing correction method applying significance threshold of 0.05 (Raudvere et al., 2019).

## Results

### rs3784711, rs7175517 and rs4776970 Associated With BMI in Chinese Children

First, to explore which genetic variants potentially have causal relationships with overweight/obesity. based on the sample size and estimated statistical power (β ≤ 0.25), six variants (MAF > 0.10 in the Chinese population) were selected for validation in Chinese children. The basic characteristics of the six variants are listed in Supplementary Table S1. Four variants, rs16951006, rs3784711, rs7175517 and rs4776970, were significantly associated with BMI even after Bonferroni correction, as shown in Table 1 (all *p* < 0.0083, Bonferroni corrected for 6 SNPs). Additionally, the associations among the 6 variants with overweight/obesity were conducted (Supplementary Table S2). All the aforementioned variants remained statistically significant even after Bonferroni correction (all *p* < 0.0083), except for rs16951006. SNP rs7175517 had the lowest *p* value among six SNPs in BMI association analyses, and similar results were detected with central obesity phenotypes in Chinese children (Supplementary Table S3) and the United Kingdom population (Supplementary Table S4).

**TABLE 1:** Association of *MAP2K5* genetic variants and BMI in Chinese children.

Gene	SNPs	BMI (*N* = 2030)		
		β^a^	SE	*p*
*MAP2K5*	rs11636408	0.25	0.13	0.051
*MAP2K5*	rs16951006	0.46	0.15	0.002^b^
*MAP2K5*	rs8037318	−0.06	0.15	0.676
*MAP2K5*	rs3784711	0.37	0.14	0.007^b^
*MAP2K5*	rs7175517	0.48	0.13	1.43 × 10^−4^ ^b^
*MAP2K5*	rs4776970	0.48	0.15	0.001^b^

SE: standard error.

a Effect sizes and *p* values were estimated under an additive genetic model adjusted for age, sex and study group.

b means the *p* value < the Bonferroni correction *p* value (*p* = 0.05/6 = 0.008).

### rs7175517 and rs4776970 Associated With BMI in the United Kingdom Population

**FIGURE 1:**
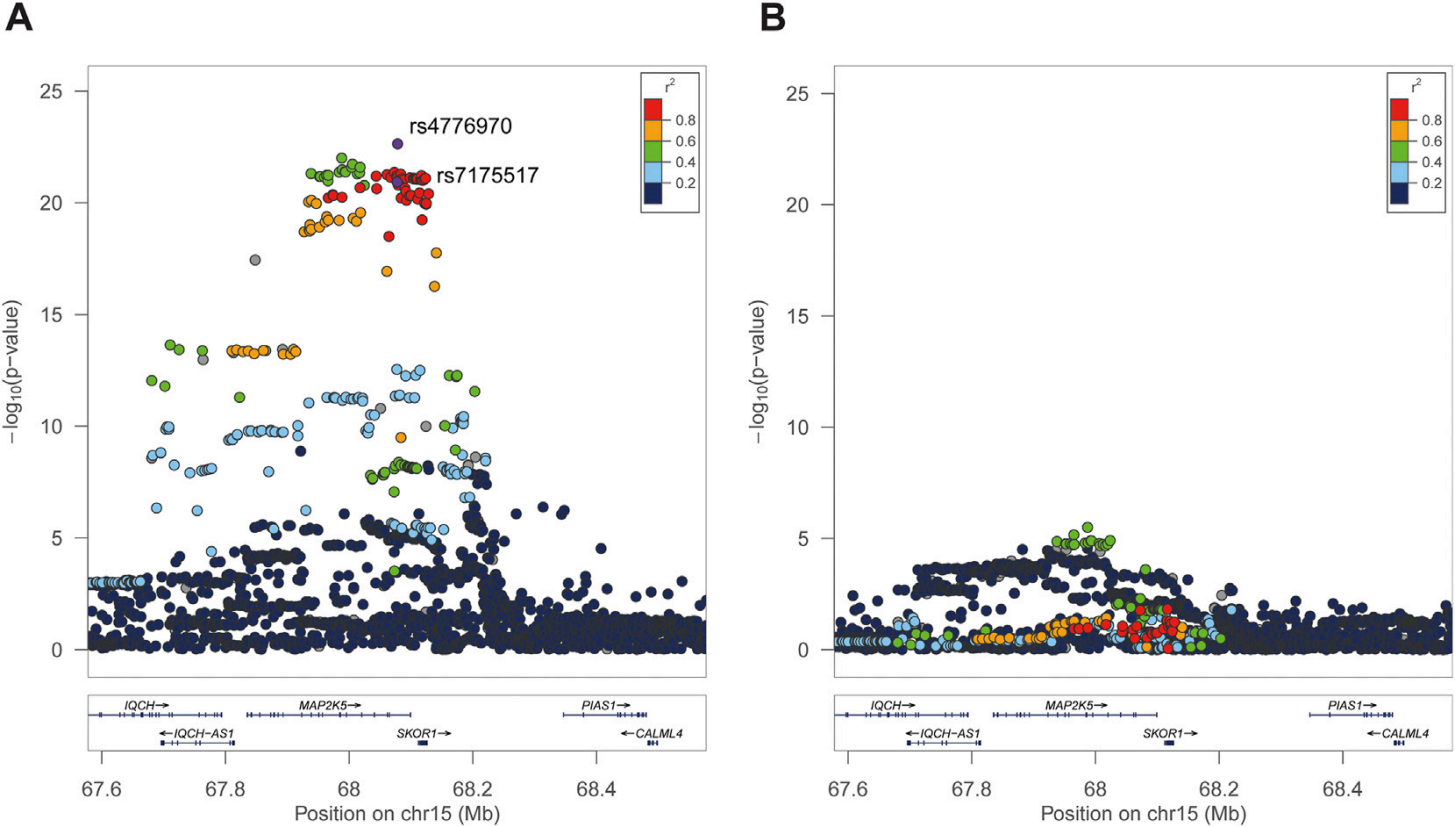
Regional association plots of *MAP2K5* loci independently associated with BMI in UKB. The *Y*-axis represents the *p* value on a -log10 scale, and the *X*-axis indicates the genetic variant localization. The *r* ^*2*^ was calculated based on rs7175517. **(A)**, The strongest association was SNP rs4776970 and rs7175517 via forward stepwise regression analysis. **(B),** After the condition for SNP rs7175517 and no other significant variant were pinpointed. The extent of linkage disequilibrium for all SNPs with rs7175517 is indicated by red colors.

Second, forward stepwise linear regressions were conducted between all selected genetic variants of *MAP2K5* and BMI. In total, 295 SNPs were significantly associated with BMI (all *p* < 5 × 10^−8^). Notably, rs4776970 (T > A, β = 0.024, *p* = 2.33 × 10^−23^) and rs7175517 (G > A, β = 0.027, *p* = 1.16 × 10^−21^) were significantly associated with BMI (Figure 1A). Notably, the effect size of rs7175517 was much higher in Chinese children (β = 0.48), which was almost 18 times as large as the value in the United Kingdom population (β = 0.027). After conditioning on either rs4776970 or rs7175517, no variant in the *MAP2K5* region reached the predefined significance threshold [*p* < 5 × 10^−8^, Figure 1B (conditioning on rs7175517) and Supplementary Figure S1B (conditioning on rs477690)].

### rs7175517 Remained the Association With BMI Through Colocalization of the eQTL and GWAS Associations for Genetic Variances of *MAP2K5*

**FIGURE 2:**
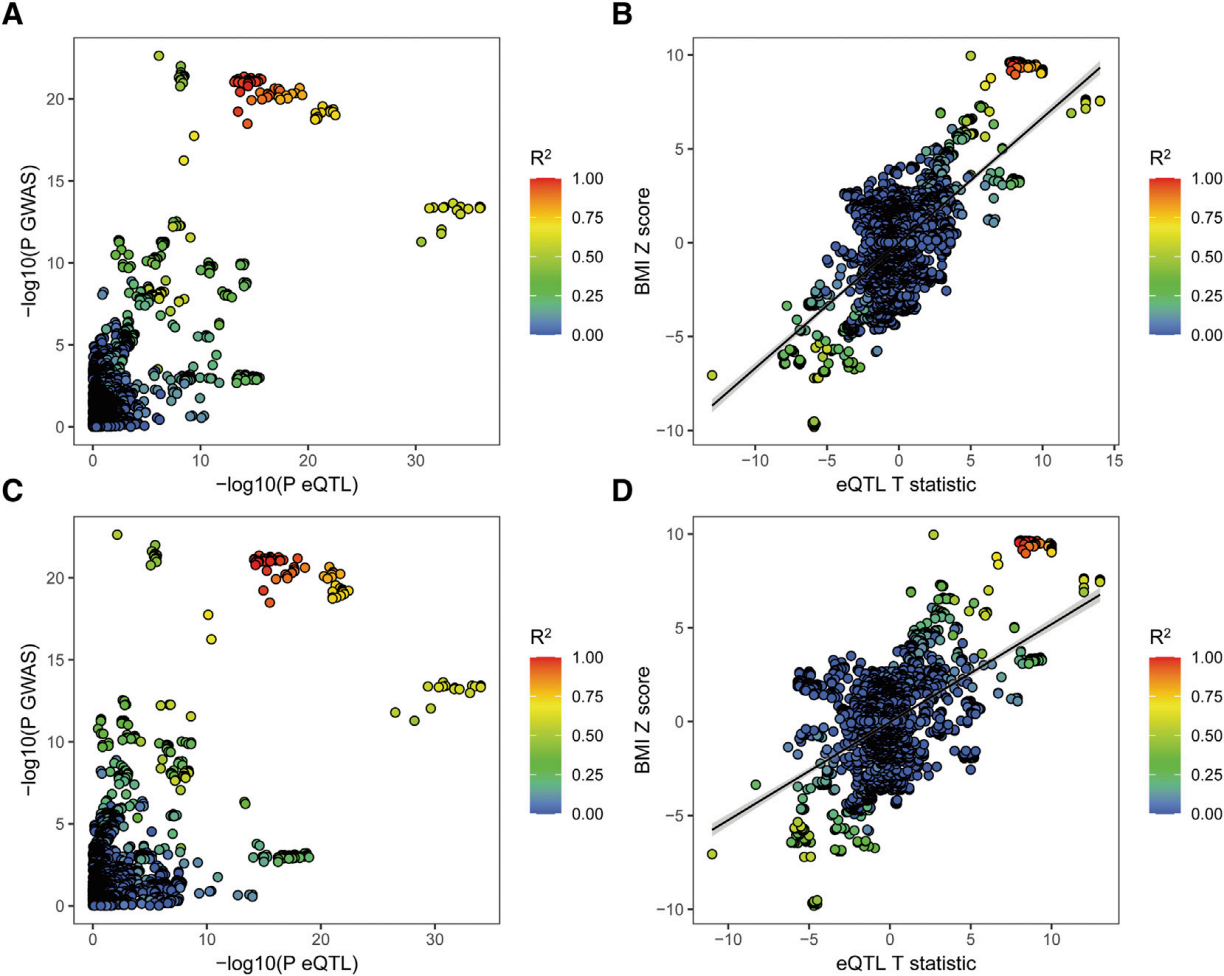
Colocalization of the eQTL and GWAS associations for *MAP2K5*. **(A),** Scatterplot shows the overlap of GWAS and eQTL associations for genetic variants of *MAP2K5* in subcutaneous adipose tissue. The *Y*-axis represents the GWAS *p* value on a -log10 scale for BMIThe *X*-axis represents the subcutaneous adipose tissue eQTL *p* value on a -log10 scale for *MAP2K5.* **(B)**, Scatterplot shows SNP associations between BMI and *MAP2K5* expression in subcutaneous adipose tissue. The *Y*-axis represents variants associated with BMI risk (*Z* score). The *X*-axis shows eQTLs associated with *MAP2K5* gene expression (t statistic). **(C)**, Scatterplot shows the overlap of GWAS and eQTL associations for genetic variants of *MAP2K5* in whole blood. The *Y*-axis represents the GWAS *p* value on a -log10 scale for BMI. The *X*-axis represents the whole blood eQTL *p* value on a -log10 scale for *MAP2K5*. **(D)**, Scatterplot shows SNP associations between BMI and *MAP2K5* expression in whole blood. The *Y*-axis represents variants associated with BMI risk (*Z* score). The *X*-axis shows eQTLs associated with *MAP2K5* gene expression (t statistic). The extent of linkage disequilibrium for all SNPs with rs7175517 is indicated by red colors.

To gain insight into the leading genetic variants in *MAP2K5* related to BMI, colocalization of Genotype-Tissue Expression (GTEx) and GWAS data was conducted in subcutaneous adipose and whole blood eQTLs (Figure 2). SNPs in strong LD with rs7175517 have more favorable associations with BMI both in subcutaneous adipose and whole blood tissues, individually (Figures 2A,C). The variants located within 1 Mb of rs7175517 increased the expression of *MAP2K5* and BMI in subcutaneous adipose and whole blood tissues, individually (Figures 2B,D). No such prominent result was detected for rs4776970.

### rs7175517 Demonstrated the Prominent Functional Properties *In-Silico* Functional Annotation and Experimental Verifications

**FIGURE 3:**
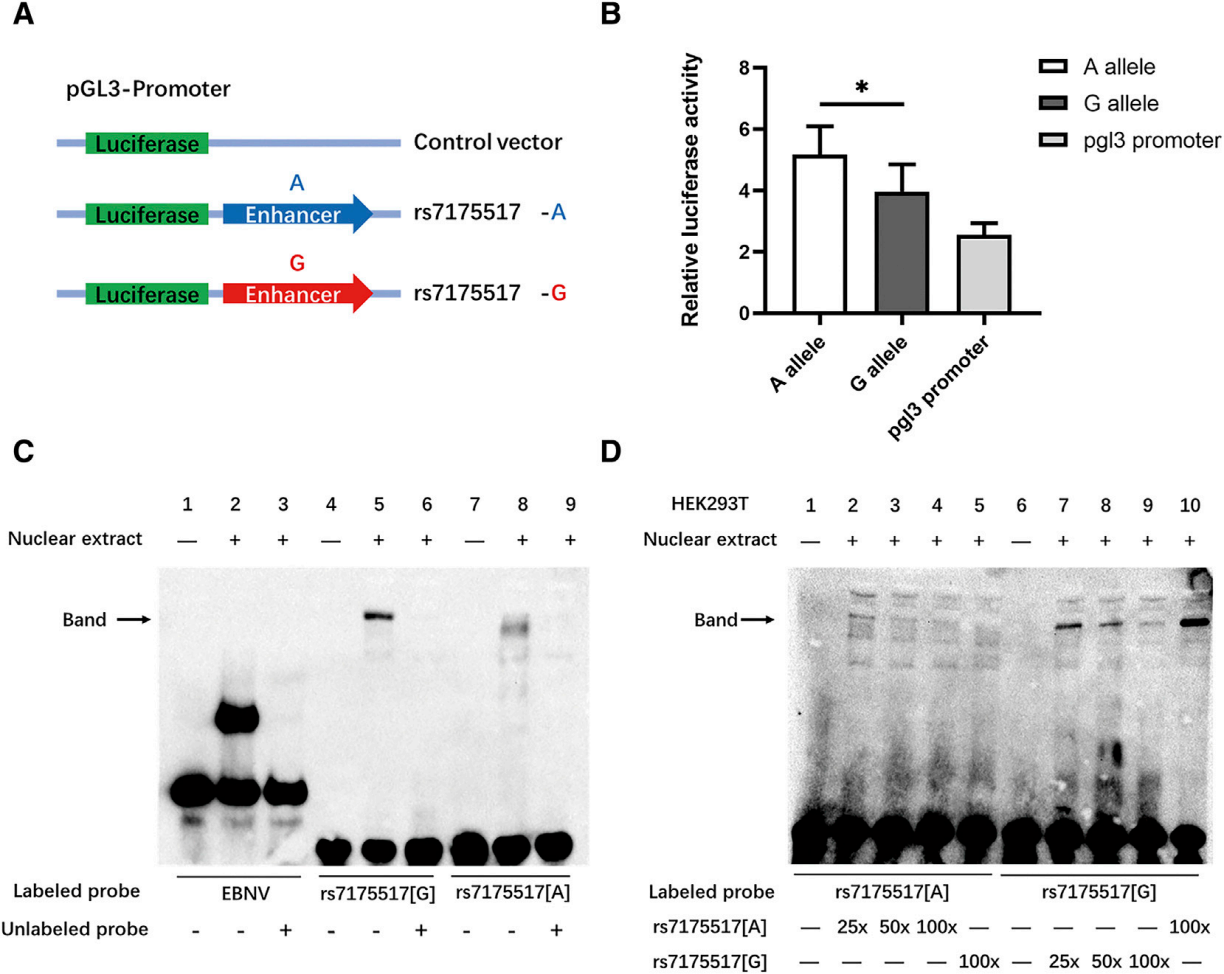
G allele of rs7175517 reduced the expression of MEK5 via binding with more inhibitor. **(A)**, Luciferase reporter assay for the region encompassing rs7175517. Each allele-specific construct contained 500 bp upstream and 500 bp downstream of the putative mutated site. All constructs were introduced into the pGL3-promoter luciferase reporter vectors. **(B)**, All constructs as indicated (100 ng per well into 24-well plates) were transiently transfected into human embryo kidney 293T (HEK293T) cells in triplicate for 48 h. **(C)**, EMSA with biotin-labeled oligonucleotides contained the rs7175517 G allele or rs7175517 A allele, and nuclei were extracted from HEK293T cells. Lanes 1, 4 and 7 show the mobility of the corresponding labeled oligonucleotides without nuclear extracts; lanes 2, 5 and 8 show the mobility of the corresponding labeled oligonucleotides with nuclear extracts in the absence of competitor; lanes 3, 6 and 9 show the mobility of the labeled oligonucleotides with nuclear extracts in the presence of unlabeled competitor. For example, more unlabeled rs7175517 oligonucleotides were added into the premixture of biotin-labeled rs7175517 to compete for the interaction with nuclear extracts. The arrow indicates a DNA-protein complex for rs7175517. **(D)**, EMSA competition assays. Lanes 1-5 indicate the competition assay for the rs7175517 A allele, and all lanes added 80 fmol biotin-labeled oligonucleotides containing the rs7175517 A allele. Lane 1 refers to those without nuclear extracts; Lanes 2, 3 and 4 refer to those with nuclear extracts and are in the presence of 25×, 50× and 100× unlabeled oligonucleotides containing the rs7175517 A allele; lane 5 refers to those with nuclear extracts and is in the presence of 100× unlabeled oligonucleotides containing the rs7175517 G allele. With the increasing amount of unlabeled oligonucleotides, the band should slowly become shallow, particularly for adding stronger interaction alleles. Similar results were presented in lanes 6–10. For lane 10, since rs7175517 G allele had stronger interaction with nuclear extracts, even 100× rs7175517 A allele was added, no fade was observed in the band. All the experiments were repeated three times, and only one experimental result was represented for each kind of experiment. For the dual luciferase assay, Student’s *t* test was adopted to test the mean differences between different conditions, and a *p* value < 0.05 was considered statistically significant.

**FIGURE 4:**
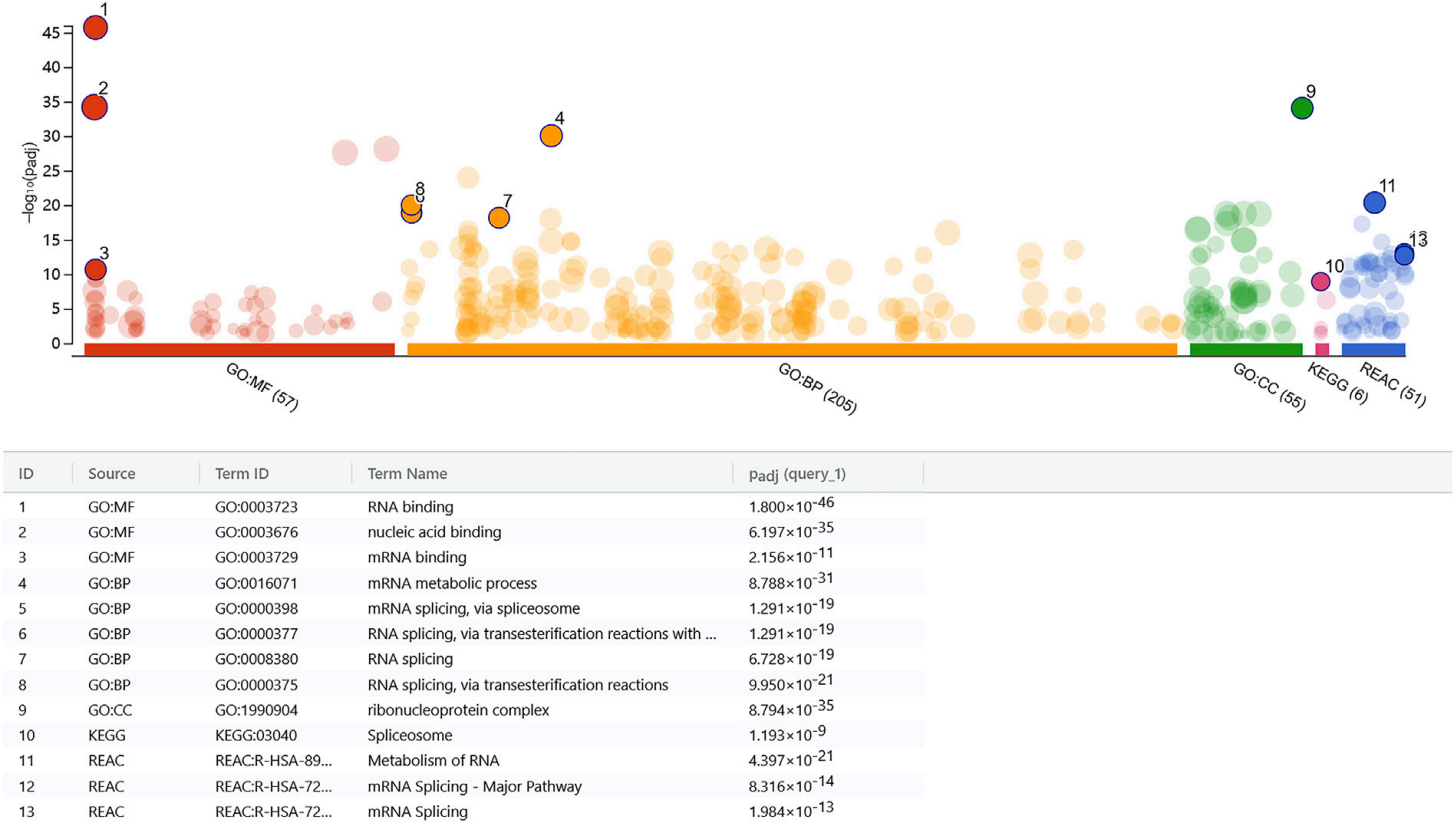
rs7175517 might involve in the RNA splicing activities. Core protein was highly expressed in the DNA pull-down assay in three databases (GO, KEGG and REAC). The GO pathway enrichment analyses, including molecular function (MF), biological process (BP) and cellular component (CC), are indicated by red, orange and green. KEGG pathway enrichment analysis is indicated by pink, and REAC pathway enrichment analysis is indicated by blue. All results indicated that co-expressed proteins were enriched in RNA splicing.

The histone modification results indicated that rs7175517 was marked with peaks of H3K4me3, H3K4me1 and H3K27ac in the ENCODE analyses from the UCSC website. We assumed that SNP rs7175517 was a strong functional variant falling within the promoter or enhancer region in comparison with rs4776970 (Supplementary Figure S2). Therefore, only rs7175517 was chosen for the functional experiments. We first conducted dual luciferase reporter assays to determine how rs7175517 affects the mRNA expression of *MAP2K5*. The results showed that the construct containing the rs7175517 [A] allele exhibited higher enhancer activity than that containing the rs7175517 [G] allele (Figures 3A,B). Consequently, electrophoretic mobility shift assay (EMSA) results indicated that the rs7175517 [G] allele preferentially banded more nuclear extracts than the rs7175517 [A] allele in HEK293T cells (Figures 3C,D). To pinpoint what kinds of nuclear proteins might bind to the G allele of rs7175517, DNA pull-down and protein mass spectrometry experiments were carried out (Supplementary Table S6). The protein mass spectrometry results were further analysed with the GO, KEGG and RACE pathways with the pathway enrichment method. The results implied that the G allele of rs7175517 may recruit more RNA spliceosomes (Figure 4).

## Discussion

Obesity is a highly complex trait caused by reciprocal genetic and environmental factors. Understanding which variant statistically significantly contributes to adipogenesis at the molecular level has been shown to be difficult. Deciphering the biological mechanisms of those significant signals in the population is a vital step to clarifying the molecular mechanism of obesity and can suggest a better prevention strategy. As the activated kinase of the ERK signaling pathway, *MAP2K5* is not only involved in the pathogenesis of adipose tissue but also plays a critical role in protecting cells from stress-induced apoptosis, neuronal survival, cardiac development and angiogenesis (Paudel et al., 2021). Fine mapping of the *MAP2K5* gene region could provide us with a panorama picture of the associations between full genetic variants located in *MAP2K5* and BMI. In the present study, fine mapping of *MAP2K5* was conducted in 2,030 Chinese children and further validated in a 264,838 United Kingdom population. Furthermore, *in-silico* functional annotation and *in vitro* experiments were consequentially carried out. All results revealed that rs7175517 of *MAP2K5* was functionally correlated with obesity in both Chinese and United Kingdom populations. The possible molecular mechanism was that the G allele of rs7175517 bound with more spliceosomes, sequentially inhibited the expression of MEK5 and then triggered more adipogenesis.

The rs2241423 SNP has very strong LD with rs7175517 (*r* ^2^ = 0.99) and is preferentially selected in most obesity-related GWAS or candidate genetic variant studies. However, no signal for rs2241423 was detected in *in-silico* functional analyses in the present study. Therefore, no further experiments were conducted. It is worth mentioning that previous research indicated that rs2241423 had a stronger effect size on obesity in Chinese children than in the Caucasian population (Wang et al., 2016). Similar trends were observed in the present study, and SNP rs7175517 had a stronger effect size on BMI in Chinese children than in the United Kingdom population, which further evidenced that *MAP2K5* had trans-ethnic differences in adipogenesis.

Making the results from our dual luciferase assay and EMAS assay together, the G allele of rs7175517 reduced the expression of *MAP2K5* and bound to more nuclear proteins. Intriguingly, our findings were akin to the research of Pan et al. (2018), who identified that the C allele of rs4776984 (high LD with rs7175517, *r* ^2^ = 0.97) increased nuclear protein binding in an allele-specific way via EMSA experiments as well. Consistently, both EMSA results indicated that nuclear proteins might interact with this region and suppress the expression of *MAP2K5*, which further triggered adipogenesis. The recent research by Joslin *et al.* revealed that rs4776984 was associated with obesity via enhancer modulating variant analyses (Joslin et al., 2021). All that information indicated that this region is highly related to BMI due to high LD among those SNPs.

Our supershift assay did not find any significant transcription factor bound to the rs7175517 region. Similar to the research conducted by Pan et al., positive transcription factors in the regulation of adipogenesis, such as CCAAT/enhancer binding protein beta (*CEBPB*) and peroxisome proliferator-activated receptor gamma (*PPARG*), were predicted to interact with the rs4776984 region in adipocytes (Moseti et al., 2016). However, none of them was evidenced by the supershift assay (Pan et al., 2018). This phenomenon might occur since there is a complex of transcription factors that bind to rs7175517 rather than a single transcription factor. In the present study, whole blood tissue also indicated that the expression of MEK5 was related to BMI. Since MEK5 was not only expressed in adipocytes, it was ubiquitously expressed in all types of cells. Therefore, it may play an important role in adipogenesis in other types of cells. For example, MEK5 involved in adipogenesis was evidenced in mouse embryonic fibroblasts via the MEK5-ERK5 signaling pathway (Zhu et al., 2014). Additionally, Cristea’s research revealed that MEK5-ERK5 participated in lipid metabolism in small-cell lung cancer through the cholesterol synthesis pathway by regulating sterol regulatory element binding protein (SREBP) (Cristea et al., 2020). All these results indicated that MEK5-ERK5 may be involved in metabolic and adipogenesis pathways throughout the whole body.

In addition, our results preferentially indicated that rs7175517 might bind to certain transcription factors that could regulate RNA splicing or expression with pathway enrichment analyses. The GO, KEGG and REAC pathway enrichment analyses all pinpointed that rs7175517 interacted with RNA spliceosomes. As the G allele of rs7175517 reduced the expression of MEK5 in the dual luciferase assay and bound with more nuclear proteins in EMSA, we speculated that certain RNAs regulating transcription factors bind to the allele-specific region of rs7175517 and modulate the expression of MEK5. Consequently, the MEK5-ERK5 signaling pathway was downregulated and triggered adiposity accumulation. In fact, the *MPA2K5*-encoded protein has two isoforms: MEK5α (50 kDa) and MEK5β (40 kDa). MEK5α is mainly expressed in the liver and brain and is particulate, while MEK5β is ubiquitously expressed and primarily cytosolic (English et al., 1995). MEK5β lacks an extended N-terminus that is present in MEK5α. The N-terminus of MEK5α is the docking site for ERK5, and MEK5α is a stronger activator of ERK5 than MEK5β (Seyfried et al., 2005). However, the specific function of MEK5β is not yet clear. Therefore, we presumed that certain RNA splicing regulators adjusted the expression of MEK5α and MEK5β to coordinate the activation of the ERK signaling pathway in adipogenesis.

In summary, we first implemented a fine mapping method to gain a comprehensive view of the associations between the *MAP2K5* gene region and BMI. The putative SNPs were further evidenced by *in vitro* experiments. Finally, we identified rs7175517 as the leading variant associated with obesity. However, the present study only included Chinese children with a limited sample size, so further validation studies with larger sample sizes and various places in China are warranted.

Overall, the present study deepened our understanding of *MAP2K5* adipogenesis throughout the whole genetic region and provided a possible target for future obesity intervention or therapy. However, to gain insight into the function of different isoforms in adipogenesis, more precisely designed molecular experiments should be conducted. In particular, *in vitro* experiments on the interaction between genetic variants (high LD variants) should be carried out to address which SNPs interact with what kind of transcription factors and how the MEK5-ERK5 signaling pathway is modulated in different tissues of the whole body.

## Conclusion

We fine mapped the *MAP2K5* region and identified SNP rs7175517 of the MAP2K5 gene as the putative causal variant associated with BMI. The results deepened our understanding of the adipogenesis of *MAP2K5* throughout the whole genetic region and provided a possible target for future obesity intervention or therapy.

## Data Availability

The original contributions presented in the study are included in the article/Supplementary Material, further inquiries can be directed to the corresponding author.

## Abbreviations

BMI, body mass index; eQTL, expression quantitative trait locus; GWAS, genome-wide association study; MAP2K5, mitogen-activated protein kinase kinase 5; MEK, MAPK/extreacellular signal-regulated kinase (ERK) kinase; UKB, United Kingdom biobank; SNP, single nucleotide polymorphism.
