# Supplementary material for: Fine Mapping of the MAP2K5 Region Identified rs7175517 as a Causal Variant Related to BMI in China and the United Kingdom Populations
Source: Front Genet. 2022 Mar 16;13:838685. doi: 10.3389/fgene.2022.838685 (PMC8967323; doi:10.3389/fgene.2022.838685)
Supplement: Supplementary file 1 [file DataSheet1.docx]

**Figure S1 Regional association plots of *MAP2K5* loci independently associated with BMI conditioning on rs4776970**

The Y-axis represents the P value on a -log10 scale, and the X-axis indicates the genetic variant localization. The r^2^ was calculated based on rs477690. (A) The strongest association was SNP rs4776970 (β = -0.12; *P* = 3.47 × 10^-18^) via forward stepwise regression analysis. (B) After the condition for SNP rs4776970, no other significant variant was pinpointed. The extent of linkage disequilibrium for all SNPs with rs7175517 is indicated by red colors.





**Figure S2 Functional annotation for rs7175517 and its related SNPs in ENCODE and Roadmap**

**
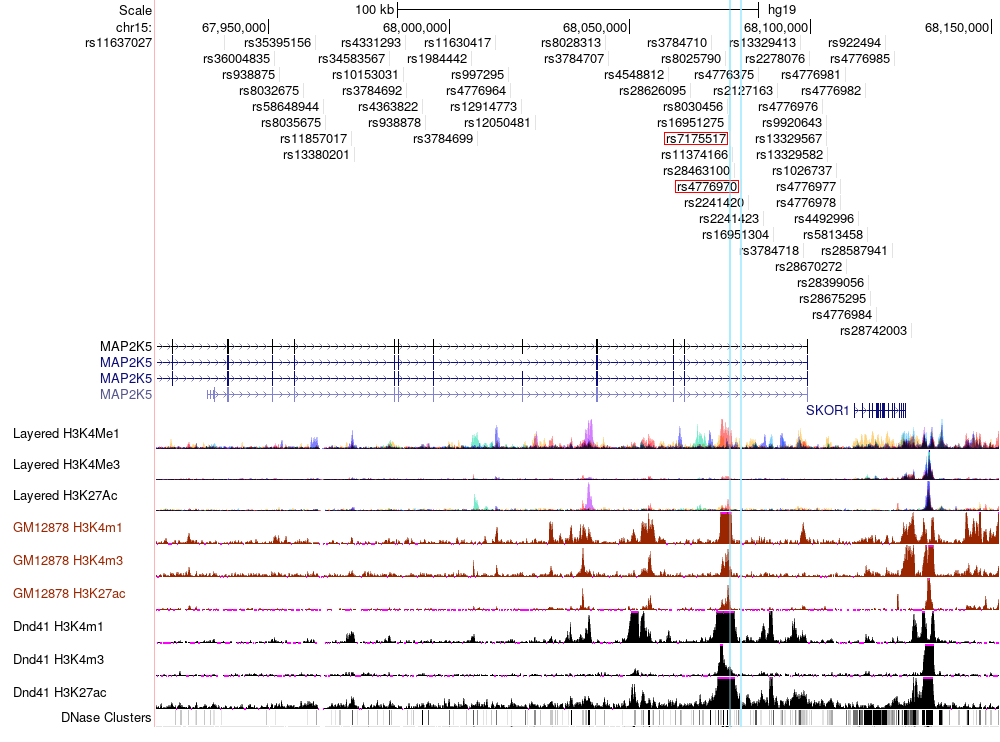
**

**Table S1 Basic genotyping information and Hardy-Weinberg test in Chinese children**

| Gene | SNP | Position | Other selected  high LD variants | Effect allele | Alternative allele | HWE-*P* value | EAF^a^ | EAF^b^ | Call rates |
| --- | --- | --- | --- | --- | --- | --- | --- | --- | --- |
|  |  |  |  |  |  |  |  |  |  |
| *MAP2K5* | rs11636408 | 15:67860568 (intron_variant) |  | G | A | 0.403 | 0.429 | 0.413 | 99.90% |
| *MAP2K5* | rs16951006 | 15:67863112 (intron_variant) |  | G | A | 1 | 0.214 | 0.184 | 99.90% |
| *MAP2K5* | rs8037318 | 15:679832327 (intron_variant) |  | G | C | 0.332 | 0.22 | 0.257 | 99.40% |
| *MAP2K5* | rs3784711 | 15:68072527 (intron_variant) | rs6494696(*r*^2^=0.952) | T | C | 0.408 | 0.304 | 0.296 | 99.90% |
| *MAP2K5* | rs7175517 | 15:68077630 (intron_variant) | rs2241423(*r*^2^=0.981), rs2278076(*r*^2^=0.991) | A | G | 0.6 | 0.409 | 0.379 | 99.90% |
|  |  |  |  |  |  |  |  |  |  |
|  |  |  |  |  |  |  |  |  |  |
| *MAP2K5* | rs4776970 | 15:68080886 (intron_variant) |  | A | T | 0.341 | 0.244 | 0.199 | 99.70% |

Position: GRCh37; HWE Hardy-Weinberg equilibrium (in the normal-weight group), EAF effect allele frequency

^a^ Effect allele frequency of the normal-weight group in the present study.

^b^ Effect allele frequency from CHB data from the 1000 Genomes Project.

**Table S2 Associations of *MAP2K5* genetic variants with overweight/obesity in Chinese children**

|  | normal-weight vs  Overweight | |  | normal-weight vs  Obesity | |  | normal-weight vs.  Severely obese | |
| --- | --- | --- | --- | --- | --- | --- | --- | --- |
| SNP | OR (95%CI) | *P* value |  | OR (95%CI) | *P* value |  | OR (95%CI) | *P* value |
| rs11636408 | 1.09(0.92-1.28) | 0.335 |  | 1.15(0.97-1.36) | 0.107 |  | 1.18(0.98-1.43) | 0.081 |
| rs16951006 | 1.12(0.92-1.37) | 0.267 |  | **1.31(1.07-1.60)** | **0.009** |  | **1.34(1.07-1.67)** | **0.010** |
| rs8037318 | 1.06(0.87-1.29) | 0.564 |  | 0.92(0.75-1.13) | 0.442 |  | 0.99(0.79-1.24) | 0.903 |
| rs3784711 | **1.28(1.07-1.53)** | **0.008^*^** |  | **1.29(1.07-1.55)** | **0.008^*^** |  | **1.36(1.11-1.68)** | **0.003^*^** |
| rs7175517 | **1.28(1.08-1.52)** | **0.005^*^** |  | **1.31(1.11-1.56)** | **0.002^*^** |  | **1.42(1.17-1.72)** | **3.59×10^-4*^** |
| rs4776970^a^ | 1.19(0.98-1.44) | 0.083 |  | **1.32(1.09-1.61)** | **0.006^*^** |  | **1.38(1.11-1.71)** | **0.003^*^** |

Effect sizes and *P* values were estimated under an additive genetic model adjusted for age, sex and study group.

^*^P value ≤ Bonferroni corrected *P*=0.008

| **Table S3 Association of *MAP2K5* genetic variants with central obesity in Chinese children** | | | | | | | | | | | | | | | | |  |
| --- | --- | --- | --- | --- | --- | --- | --- | --- | --- | --- | --- | --- | --- | --- | --- | --- | --- |
| Gene | SNP | Waist circumference (cm, n=2017) | | |  | Hip circumference (cm, n=2016) | | |  | WHR (n=2016) | | |  | WHtR (n=2017) | | | |
|  |  | β^*^ | SE | *P* |  | β^*^ | SE | *P* |  | β^*^ | SE | *P* |  | β^*^ | SE | *P* | |
| *MAP2K5* | rs11636408 | **0.65** | **0.33** | **0.048** |  | 0.33 | 0.26 | 0.199 |  | **0.0039** | **0.0018** | **0.029** |  | **0.0047** | **0.0019** | **0.012** | |
| *MAP2K5* | rs16951006 | **1.14** | **0.39** | **0.003** |  | **0.65** | **0.31** | **0.034** |  | **0.0060** | **0.0021** | **0.005** |  | **0.0076** | **0.0022** | **6.57×10^-4#^** | |
| *MAP2K5* | rs8037318 | -0.05 | 0.40 | 0.901 |  | -0.05 | 0.31 | 0.881 |  | 0.0002 | 0.0022 | 0.912 |  | -0.0003 | 0.0023 | 0.880 | |
| *MAP2K5* | rs3784711 | **0.99** | **0.36** | **0.005** |  | **0.80** | **0.28** | **0.005** |  | 0.0038 | 0.0020 | 0.054 |  | **0.0056** | **0.0020** | **0.006^#^** | |
| *MAP2K5* | rs7175517 | **1.19** | **0.33** | **3.28×10^-4^** |  | **0.84** | **0.26** | **0.001** |  | **0.0050** | **0.0018** | **0.006** |  | **0.0073** | **0.0019** | **1.28×10^-4#^** | |
| MAP2K5 | rs4776970 | **1.25** | **0.38** | **0.001** |  | **0.71** | **0.30** | **0.019** |  | **0.0063** | **0.0021** | **0.003** |  | **0.0079** | **0.0022** | **3.11×10^-4#^** | |
| WHR: Weight-hip circumference; WHtR, Waist-height ratio | | | | | | | | | | | | | | | | |  |
| *Effect sizes and *P* values were estimated under additive genetic model adjusted for age, sex and study group. | | | | | | | | | | | | | | | | |  |

^#^P value ≤ Bonferroni corrected *P*=0.008

**Table S4 Association of *MAP2K5* genetic variants with waist circumference in the UK population**

| SNP | β | SE | *P* |
| --- | --- | --- | --- |
| rs7175517_A | 0.279 | 0.031 | **< 2×10^-16^** |
| rs4776970_A | 0.227 | 0.027 | **< 2×10^-16^** |
| rs3784711_T | 0.111 | 0.027 | **3.07×10^-5^** |
| rs16951006_G | 0.153 | 0.045 | **6.94×10^-5^** |

Effect sizes and *P* values were estimated under an additive genetic model adjusted for age, sex, income, educational attainment, genotype measurement batch and PCAs.

**Table S5 Oligonucleotides used in EMSAs.**

| Name | Sequence (5’-3’)^a^ |
| --- | --- |
| rs7175517_A ^BIO^ | ^BIO^TTGTGCCAGGATATAAATCAAGCCT |
| rs7175517_G ^BIO^ | ^BIO^TTGTGCCAGGATGTAAATCAAGCCT |
| rs7175517_A | TTGTGCCAGGATATAAATCAAGCCT |
| rs7175517_G | TTGTGCCAGGATGTAAATCAAGCCT |

^a^ BIO: 5’ biotinylation on both the sense and antisense strands of the duplex

Table S6 Mass-Spectrometry results of the DNA-Pull Down experiments.

| **First protein** | **Gene name** | **Description** | **Peptides** | **Unique peptides** | **Sequence coverage [%]** | **Unique sequence coverage [%]** | **Mol. weight [kDa]** | **Sequence length** |
| --- | --- | --- | --- | --- | --- | --- | --- | --- |
| P09874 | PARP1 | Poly [ADP-ribose] polymerase 1 | 30 | 30 | 27 | 27 | 113.08 | 1014 |
| A0A087X0X3 | HNRNPM | Heterogeneous nuclear ribonucleoprotein M | 17 | 17 | 23.2 | 23.2 | 77.569 | 730 |
| A0A0C4DGA6 | HLTF | Helicase-like transcription factor | 16 | 16 | 14.4 | 14.4 | 113.86 | 1008 |
| P27694 | RPA1 | Replication protein A 70 kDa DNA-binding subunit | 13 | 13 | 22.6 | 22.6 | 68.137 | 616 |
| P08670 | VIM | Vimentin | 12 | 10 | 26.6 | 23 | 53.651 | 466 |
| F8W6I7 | HNRNPA1 | Heterogeneous nuclear ribonucleoprotein A1 | 11 | 11 | 33.2 | 33.2 | 33.155 | 307 |
| P11498 | PC | Pyruvate carboxylase, mitochondrial | 10 | 10 | 9 | 9 | 129.63 | 1178 |
| P19338 | NCL | Nucleolin | 9 | 9 | 12.1 | 12.1 | 76.613 | 710 |
| P63261 | ACTG1 | Actin, cytoplasmic 2 | 8 | 2 | 21.3 | 6.1 | 41.792 | 375 |
| P02768 | ALB | Serum albumin | 8 | 5 | 12.8 | 8.9 | 69.366 | 609 |
| P63267 | ACTG2 | Actin, gamma-enteric smooth muscle | 7 | 1 | 17.8 | 2.7 | 41.876 | 376 |
| P22626 | HNRNPA2B1 | Heterogeneous nuclear ribonucleoproteins A2/B1 | 7 | 7 | 21 | 21 | 37.429 | 353 |
| Q00839-2 | HNRNPU | Isoform Short of Heterogeneous nuclear ribonucleoprotein U | 7 | 7 | 8.7 | 8.7 | 88.979 | 806 |
| P16403 | HIST1H1C | Histone H1.2 | 6 | 2 | 22.5 | 9.4 | 21.364 | 213 |
| P16402 | HIST1H1D | Histone H1.3 | 6 | 1 | 22.6 | 7.2 | 22.35 | 221 |
| P10412 | HIST1H1E | Histone H1.4 | 6 | 1 | 24.7 | 9.1 | 21.865 | 219 |
| P09429 | HMGB1 | High mobility group protein B1 | 6 | 5 | 30.2 | 26 | 24.893 | 215 |
| P61978-3 | HNRNPK | Isoform 3 of Heterogeneous nuclear ribonucleoprotein K | 6 | 6 | 15 | 15 | 48.562 | 440 |
| P78527-2 | PRKDC | Isoform 2 of DNA-dependent protein kinase catalytic subunit | 6 | 6 | 1.5 | 1.5 | 465.5 | 4097 |
| Q9NR30-2 | DDX21 | Isoform 2 of Nucleolar RNA helicase 2 | 5 | 5 | 7.8 | 7.8 | 79.656 | 715 |
| A0A087WVQ9 | EEF1A1 | Elongation factor 1-alpha 1 | 5 | 5 | 10 | 10 | 47.883 | 441 |
| I3L1L3 | MYBBP1A | Myb-binding protein 1A (Fragment) | 5 | 5 | 4.2 | 4.2 | 140.13 | 1252 |
| P06748-3 | NPM1 | Isoform 3 of Nucleophosmin | 5 | 5 | 14.7 | 14.7 | 28.4 | 259 |
| Q5JP53 | TUBB | Tubulin beta chain | 5 | 1 | 12.7 | 2.8 | 47.766 | 426 |
| P68371 | TUBB4B | Tubulin beta-4B chain | 5 | 1 | 12.1 | 2.7 | 49.83 | 445 |
| P13010 | XRCC5 | X-ray repair cross-complementing protein 5 | 5 | 5 | 5.3 | 5.3 | 82.704 | 732 |
| P15924 | DSP | Desmoplakin | 4 | 4 | 1.4 | 1.4 | 331.77 | 2871 |
| P78347-2 | GTF2I | Isoform 2 of General transcription factor II-I | 4 | 4 | 4.8 | 4.8 | 107.97 | 957 |
| P26583 | HMGB2 | High mobility group protein B2 | 4 | 3 | 16.7 | 12.4 | 24.033 | 209 |
| Q9HCC0-2 | MCCC2 | Isoform 2 of Methylcrotonoyl-CoA carboxylase beta chain, mitochondrial | 4 | 4 | 8 | 8 | 57.518 | 525 |
| A6NLN1 | PTBP1 | Polypyrimidine tract binding protein 1, isoform CRA_b | 4 | 4 | 8 | 8 | 56.51 | 527 |
| B5MDF5 | RAN | GTP-binding nuclear protein Ran | 4 | 4 | 18.5 | 18.5 | 26.224 | 233 |
| Q96PK6 | RBM14 | RNA-binding protein 14 | 4 | 4 | 7 | 7 | 69.491 | 669 |
| P38159-2 | RBMX | Isoform 2 of RNA-binding motif protein, X chromosome | 4 | 4 | 10.3 | 10.3 | 40.846 | 378 |
| P62913 | RPL11 | 60S ribosomal protein L11 | 4 | 4 | 23.6 | 23.6 | 20.252 | 178 |
| P26373 | RPL13 | 60S ribosomal protein L13 | 4 | 4 | 21.3 | 21.3 | 24.261 | 211 |
| Q02878 | RPL6 | 60S ribosomal protein L6 | 4 | 4 | 11.5 | 11.5 | 32.728 | 288 |
| A0A0G2JLD8 | SSBP1 | Single-stranded DNA-binding protein, mitochondrial (Fragment) | 4 | 4 | 26.9 | 26.9 | 15.598 | 134 |
| H0YMM1 | ANXA2 | Annexin (Fragment) | 3 | 3 | 18.8 | 18.8 | 16.458 | 149 |
| H3BLZ8 | DDX17 | Probable ATP-dependent RNA helicase DDX17 | 3 | 2 | 5.2 | 3.6 | 80.439 | 731 |
| J3KTA4 | DDX5 | Probable ATP-dependent RNA helicase DDX5 | 3 | 2 | 4.7 | 2.8 | 69.086 | 614 |
| G3V555 | HNRNPC | Heterogeneous nuclear ribonucleoproteins C1/C2 (Fragment) | 3 | 3 | 14.3 | 14.3 | 19.178 | 175 |
| D6RF44 | HNRNPD | Heterogeneous nuclear ribonucleoprotein D0 (Fragment) | 3 | 3 | 32.4 | 32.4 | 12.553 | 111 |
| E9PCY7 | HNRNPH1 | Heterogeneous nuclear ribonucleoprotein H | 3 | 3 | 8.2 | 8.2 | 47.087 | 429 |
| P10809 | HSPD1 | 60 kDa heat shock protein, mitochondrial | 3 | 3 | 5.2 | 5.2 | 61.054 | 573 |
| P52948-4 | NUP98 | Isoform 4 of Nuclear pore complex protein Nup98-Nup96 | 3 | 3 | 3 | 3 | 96.073 | 920 |
| Q15365 | PCBP1 | Poly(rC)-binding protein 1 | 3 | 3 | 8.7 | 8.7 | 37.497 | 356 |
| A0A0A0MSI0 | PRDX1 | Peroxiredoxin-1 (Fragment) | 3 | 3 | 17.5 | 17.5 | 18.976 | 171 |
| A8MUD9 | RPL7 | 60S ribosomal protein L7 | 3 | 3 | 14.4 | 14.4 | 24.433 | 208 |
| P62269 | RPS18 | 40S ribosomal protein S18 | 3 | 3 | 19.1 | 19.1 | 17.718 | 152 |
| Q5JR95 | RPS8 | 40S ribosomal protein S8 | 3 | 3 | 15.4 | 15.4 | 21.879 | 188 |
| P11387 | TOP1 | DNA topoisomerase 1 | 3 | 3 | 3.5 | 3.5 | 90.725 | 765 |
| H0Y449 | YBX1 | Nuclease-sensitive element-binding protein 1 (Fragment) | 3 | 3 | 15.2 | 15.2 | 41.905 | 374 |
| Q13085-3 | ACACA | Isoform 3 of Acetyl-CoA carboxylase 1 | 2 | 2 | 0.9 | 0.9 | 257.24 | 2268 |
| G3V5Q1 | APEX1 | DNA-(apurinic or apyrimidinic site) lyase (Fragment) | 2 | 2 | 8.3 | 8.3 | 27.125 | 242 |
| E9PP50 | CFL1 | Cofilin-1 (Fragment) | 2 | 2 | 14.5 | 14.5 | 17.777 | 159 |
| K7EQ02 | DAZAP1 | DAZ-associated protein 1 (Fragment) | 2 | 2 | 8 | 8 | 35.02 | 327 |
| P81605 | DCD | Dermcidin | 2 | 2 | 20 | 20 | 11.284 | 110 |
| Q08211 | DHX9 | ATP-dependent RNA helicase A | 2 | 2 | 1.7 | 1.7 | 140.96 | 1270 |
| P26641 | EEF1G | Elongation factor 1-gamma | 2 | 2 | 5.9 | 5.9 | 50.118 | 437 |
| P13639 | EEF2 | Elongation factor 2 | 2 | 2 | 2.3 | 2.3 | 95.337 | 858 |
| Q15717 | ELAVL1 | ELAV-like protein 1 | 2 | 2 | 6.1 | 6.1 | 36.091 | 326 |
| O14556 | GAPDHS | Glyceraldehyde-3-phosphate dehydrogenase, testis-specific | 2 | 2 | 4.4 | 4.4 | 44.501 | 408 |
| F5H2U8 | HMGA2 | High mobility group protein HMGI-C | 2 | 2 | 36.4 | 36.4 | 10.767 | 99 |
| P05114 | HMGN1 | Non-histone chromosomal protein HMG-14 | 2 | 2 | 26 | 26 | 10.659 | 100 |
| Q13151 | HNRNPA0 | Heterogeneous nuclear ribonucleoprotein A0 | 2 | 2 | 6.9 | 6.9 | 30.84 | 305 |
| P51991-2 | HNRNPA3 | Isoform 2 of Heterogeneous nuclear ribonucleoprotein A3 | 2 | 2 | 6.5 | 6.5 | 37.029 | 356 |
| D6R9P3 | HNRNPAB | Heterogeneous nuclear ribonucleoprotein A/B | 2 | 2 | 9.6 | 9.6 | 30.302 | 280 |
| A0A087WUK2 | HNRNPDL | Heterogeneous nuclear ribonucleoprotein D-like | 2 | 2 | 6.6 | 6.6 | 40.04 | 363 |
| O43390-4 | HNRNPR | Isoform 4 of Heterogeneous nuclear ribonucleoprotein R | 2 | 2 | 4.3 | 4.3 | 59.952 | 535 |
| P17066 | HSPA6 | Heat shock 70 kDa protein 6 | 2 | 1 | 3.1 | 1.7 | 71.027 | 643 |
| Q86X29-6 | LSR | Isoform 6 of Lipolysis-stimulated lipoprotein receptor | 2 | 2 | 5.1 | 5.1 | 54.498 | 493 |
| G5E9X5 | MCCC1 | Methylcrotonoyl-CoA carboxylase subunit alpha, mitochondrial | 2 | 2 | 4.6 | 4.6 | 48.216 | 434 |
| P35658-2 | NUP214 | Isoform 2 of Nuclear pore complex protein Nup214 | 2 | 2 | 1.1 | 1.1 | 212.57 | 2079 |
| F8WBI9 | PCCB | Propionyl-CoA carboxylase beta chain, mitochondrial | 2 | 2 | 6.4 | 6.4 | 43.712 | 405 |
| Q5SZU1 | PHGDH | D-3-phosphoglycerate dehydrogenase | 2 | 2 | 5.2 | 5.2 | 53.085 | 499 |
| Q5TEJ7 | RPA2 | Replication protein A 32 kDa subunit (Fragment) | 2 | 2 | 13.4 | 13.4 | 19.433 | 179 |
| P40429 | RPL13A | 60S ribosomal protein L13a | 2 | 2 | 10.8 | 10.8 | 23.577 | 203 |
| M0R3D6 | RPL18A | 60S ribosomal protein L18a (Fragment) | 2 | 2 | 16.3 | 16.3 | 16.714 | 141 |
| E9PJD9 | RPL27A | 60S ribosomal protein L27a | 2 | 2 | 25.3 | 25.3 | 10.127 | 91 |
| H0YLP6 | RPL28 | 60S ribosomal protein L28 | 2 | 2 | 24.7 | 24.7 | 9.657 | 89 |
| P49207 | RPL34 | 60S ribosomal protein L34 | 2 | 2 | 18.8 | 18.8 | 13.293 | 117 |
| H3BM89 | RPL4 | 60S ribosomal protein L4 | 2 | 2 | 5.7 | 5.7 | 37.657 | 333 |
| P62263 | RPS14 | 40S ribosomal protein S14 | 2 | 2 | 15.9 | 15.9 | 16.273 | 151 |
| P39019 | RPS19 | 40S ribosomal protein S19 | 2 | 2 | 13.1 | 13.1 | 16.06 | 145 |
| E9PPU1 | RPS3 | 40S ribosomal protein S3 | 2 | 2 | 16.5 | 16.5 | 17.407 | 158 |
| B5MCP9 | RPS7 | 40S ribosomal protein S7 | 2 | 2 | 8.6 | 8.6 | 21.312 | 187 |
| Q8NC51-4 | SERBP1 | Isoform 4 of Plasminogen activator inhibitor 1 RNA-binding protein | 2 | 2 | 7 | 7 | 42.426 | 387 |
| P05141 | SLC25A5 | ADP/ATP translocase 2 | 2 | 2 | 6.4 | 6.4 | 32.852 | 298 |
| A0A087X2D0 | SRSF3 | Serine/arginine-rich-splicing factor 3 | 2 | 2 | 21.1 | 21.1 | 10.32 | 95 |
| P26368-2 | U2AF2 | Isoform 2 of Splicing factor U2AF 65 kDa subunit | 2 | 2 | 3.6 | 3.6 | 53.12 | 471 |
| B1AHC9 | XRCC6 | X-ray repair cross-complementing protein 6 | 2 | 2 | 4.8 | 4.8 | 64.283 | 559 |
| O96019-2 | ACTL6A | Isoform 2 of Actin-like protein 6A | 1 | 1 | 2.6 | 2.6 | 43.236 | 387 |
| F6THM6 | ACTN2 | Alpha-actinin-2 | 1 | 1 | 0.9 | 0.9 | 79.674 | 686 |
| E9PB61 | ALYREF | THO complex subunit 4 | 1 | 1 | 4.2 | 4.2 | 27.557 | 264 |
| C9JS07 | AMER3 | APC membrane recruitment protein 3 (Fragment) | 1 | 1 | 7.8 | 7.8 | 19.381 | 180 |
| P25311 | AZGP1 | Zinc-alpha-2-glycoprotein | 1 | 1 | 3.4 | 3.4 | 34.258 | 298 |
| J3QSX4 | BUB3 | Mitotic checkpoint protein BUB3 | 1 | 1 | 9 | 9 | 16.28 | 145 |
| Q9BXJ3 | C1QTNF4 | Complement C1q tumor necrosis factor-related protein 4 | 1 | 1 | 3 | 3 | 35.256 | 329 |
| F8WBR5 | CALM2 | Calmodulin | 1 | 1 | 20 | 20 | 7.372 | 65 |
| Q9NZT1 | CALML5 | Calmodulin-like protein 5 | 1 | 1 | 9.6 | 9.6 | 15.892 | 146 |
| B7Z4W5 | CCBL1 | Cysteine conjugate-beta lyase cytoplasmic (Glutamine transaminase K, kyneurenine aminotransferase), isoform CRA_b | 1 | 1 | 5.2 | 5.2 | 57.668 | 516 |
| E7ENZ3 | CCT5 | T-complex protein 1 subunit epsilon | 1 | 1 | 2.1 | 2.1 | 53.848 | 486 |
| P53567 | CEBPG | CCAAT/enhancer-binding protein gamma | 1 | 1 | 6.7 | 6.7 | 16.408 | 150 |
| A0A087X182 | CFAP45 | Cilia- and flagella-associated protein 45 | 1 | 1 | 2.1 | 2.1 | 50.352 | 431 |
| A0A087X1B7 | CHTOP | Chromatin target of PRMT1 protein | 1 | 1 | 17.1 | 17.1 | 8.5199 | 76 |
| Q14011 | CIRBP | Cold-inducible RNA-binding protein | 1 | 1 | 6.4 | 6.4 | 18.648 | 172 |
| Q2KHT3-2 | CLEC16A | Isoform 2 of Protein CLEC16A | 1 | 1 | 1.2 | 1.2 | 103.55 | 906 |
| Q6NT55 | CYP4F22 | Cytochrome P450 4F22 | 1 | 1 | 1.3 | 1.3 | 61.958 | 531 |
| B4E099 | DBF4B | Protein DBF4 homolog B | 1 | 1 | 6.4 | 6.4 | 16.961 | 157 |
| A0A0D9SFB3 | DDX3X | ATP-dependent RNA helicase DDX3X | 1 | 1 | 1.7 | 1.7 | 70.839 | 640 |
| H7C5N3 | DNAH12 | Dynein heavy chain 12, axonemal (Fragment) | 1 | 1 | 0.9 | 0.9 | 84.652 | 740 |
| Q02413 | DSG1 | Desmoglein-1 | 1 | 1 | 0.9 | 0.9 | 113.75 | 1049 |
| E9PN91 | EEF1D | Elongation factor 1-delta | 1 | 1 | 11.3 | 11.3 | 11.616 | 106 |
| J3KSN7 | EIF4A2 | Eukaryotic initiation factor 4A-II (Fragment) | 1 | 1 | 7.3 | 7.3 | 12.071 | 109 |
| F8W9B8 | EXOC5 | Exocyst complex component 5 | 1 | 1 | 1.7 | 1.7 | 74.34 | 643 |
| Q9NQT5-2 | EXOSC3 | Isoform 2 of Exosome complex component RRP40 | 1 | 1 | 9.1 | 9.1 | 17.247 | 164 |
| E9PI41 | EXOSC4 | Exosome complex component RRP41 (Fragment) | 1 | 1 | 3.8 | 3.8 | 28.342 | 261 |
| D6REE0 | FAM153A | Protein FAM153A (Fragment) | 1 | 1 | 66.7 | 66.7 | 2.732 | 24 |
| A0A087WUA0 | FGA | Fibrinogen alpha chain | 1 | 1 | 2.8 | 2.8 | 33.005 | 289 |
| P20930 | FLG | Filaggrin | 1 | 1 | 0.2 | 0.2 | 435.16 | 4061 |
| Q5D862 | FLG2 | Filaggrin-2 | 1 | 1 | 0.5 | 0.5 | 248.07 | 2391 |
| Q08378-4 | GOLGA3 | Isoform 3 of Golgin subfamily A member 3 | 1 | 1 | 0.6 | 0.6 | 126.13 | 1134 |
| Q6IC98-2 | GRAMD4 | Isoform 2 of GRAM domain-containing protein 4 | 1 | 1 | 9.9 | 9.9 | 10.922 | 101 |
| P07305 | H1F0 | Histone H1.0 | 1 | 1 | 6.7 | 6.7 | 20.863 | 194 |
| Q92522 | H1FX | Histone H1x | 1 | 1 | 7 | 7 | 22.487 | 213 |
| C9J0D1 | H2AFV | Histone H2A | 1 | 1 | 7.4 | 7.4 | 13.163 | 122 |
| K7EMV3 | H3F3B | Histone H3 | 1 | 1 | 7.6 | 7.6 | 10.334 | 92 |
| Q8WY65 | hCG_2019237 | HCG2019237 | 1 | 1 | 8.4 | 8.4 | 12.831 | 119 |
| U3KQK0 | HIST1H2BN | Histone H2B | 1 | 1 | 6.6 | 6.6 | 18.804 | 166 |
| P62805 | HIST1H4A | Histone H4 | 1 | 1 | 9.7 | 9.7 | 11.367 | 103 |
| P17096 | HMGA1 | High mobility group protein HMG-I/HMG-Y | 1 | 1 | 15 | 15 | 11.676 | 107 |
| E9PES6 | HMGB3 | High mobility group protein B3 (Fragment) | 1 | 1 | 8.5 | 8.5 | 17.522 | 153 |
| A0A087WZE9 | HMGN3 | High mobility group nucleosome-binding domain-containing protein 3 | 1 | 1 | 10 | 10 | 13.956 | 130 |
| B7WPG3 | HNRNPLL | Heterogeneous nuclear ribonucleoprotein L-like | 1 | 1 | 1.8 | 1.8 | 56.45 | 508 |
| P17482 | HOXB9 | Homeobox protein Hox-B9 | 1 | 1 | 5.2 | 5.2 | 28.058 | 250 |
| A0A075B6L0 | IGLC3 | Ig lambda-3 chain C regions (Fragment) | 1 | 1 | 9.4 | 9.4 | 11.208 | 106 |
| Q14643-4 | ITPR1 | Isoform 4 of Inositol 1,4,5-trisphosphate receptor type 1 | 1 | 1 | 0.3 | 0.3 | 306.77 | 2695 |
| F8W719 | ITSN2 | Intersectin-2 (Fragment) | 1 | 1 | 5.5 | 5.5 | 19.713 | 183 |
| C9JTX4 | JUP | Junction plakoglobin (Fragment) | 1 | 1 | 5.1 | 5.1 | 25.705 | 235 |
| Q07666-2 | KHDRBS1 | Isoform 2 of KH domain-containing, RNA-binding, signal transduction-associated protein 1 | 1 | 1 | 2.4 | 2.4 | 45.86 | 418 |
| M0QXW7 | KHSRP | Far upstream element-binding protein 2 (Fragment) | 1 | 1 | 11.2 | 11.2 | 12.086 | 116 |
| Q0VGE6 | KLHL17 | KLHL17 protein | 1 | 1 | 4.4 | 4.4 | 24.348 | 225 |
| Q76NI1-2 | KNDC1 | Isoform 2 of Protein very KIND | 1 | 1 | 0.7 | 0.7 | 122.64 | 1141 |
| J3QL07 | KPNA2 | Importin subunit alpha-1 | 1 | 1 | 24 | 24 | 5.5029 | 50 |
| Q14739 | LBR | Lamin-B receptor | 1 | 1 | 1.8 | 1.8 | 70.702 | 615 |
| O75829-2 | LECT1 | Isoform 2 of Leukocyte cell-derived chemotaxin 1 | 1 | 1 | 2.1 | 2.1 | 36.974 | 333 |
| Q9HBX8-1 | LGR6 | Isoform 1 of Leucine-rich repeat-containing G-protein coupled receptor 6 | 1 | 1 | 1.3 | 1.3 | 89.301 | 828 |
| P49916-4 | LIG3 | Isoform 4 of DNA ligase 3 | 1 | 1 | 1.2 | 1.2 | 95.801 | 862 |
| A0A0B4J2F2 | LOC102724428 | Protein LOC102724428 | 1 | 1 | 1.4 | 1.4 | 84.929 | 783 |
| F8W9L6 | LRMP | Lymphoid-restricted membrane protein | 1 | 1 | 2.3 | 2.3 | 38.995 | 348 |
| Q9HCI5 | MAGEE1 | Melanoma-associated antigen E1 | 1 | 1 | 0.8 | 0.8 | 103.25 | 957 |
| A8MWG7 | MAP3K19 | Mitogen-activated protein kinase kinase kinase 19 | 1 | 1 | 0.7 | 0.7 | 150.54 | 1328 |
| H3BQS2 | MAZ | Myc-associated zinc finger protein (Fragment) | 1 | 1 | 5.3 | 5.3 | 15.978 | 152 |
| F8W883 | MYO16 | Unconventional myosin-XVI | 1 | 1 | 0.6 | 0.6 | 208.84 | 1880 |
| A0A087WV29 | NAT10 | RNA cytidine acetyltransferase | 1 | 1 | 1.8 | 1.8 | 93.533 | 834 |
| J3KSB5 | NF1 | Neurofibromin (Fragment) | 1 | 1 | 0.4 | 0.4 | 186.43 | 1656 |
| S4R3C2 | NOLC1 | Nucleolar and coiled-body phosphoprotein 1 (Fragment) | 1 | 1 | 3.2 | 3.2 | 29.164 | 281 |
| F5H2G5 | NRIP2 | Nuclear receptor-interacting protein 2 (Fragment) | 1 | 1 | 4.6 | 4.6 | 19.722 | 175 |
| F5H6Y5 | NUMA1 | Nuclear mitotic apparatus protein 1 (Fragment) | 1 | 1 | 1.7 | 1.7 | 78.289 | 691 |
| A0A140T9F1 | OR12D2 | Olfactory receptor | 1 | 1 | 3.3 | 3.3 | 34.943 | 307 |
| K7ELH1 | PARD6G | Partitioning defective 6 homolog gamma (Fragment) | 1 | 1 | 16.4 | 16.4 | 6.8656 | 61 |
| A0A1B0GWA1 | PCCA | Propionyl-CoA carboxylase alpha chain, mitochondrial (Fragment) | 1 | 1 | 3.4 | 3.4 | 36.036 | 326 |
| P12273 | PIP | Prolactin-inducible protein | 1 | 1 | 7.5 | 7.5 | 16.572 | 146 |
| P08F94-2 | PKHD1 | Isoform 2 of Fibrocystin | 1 | 1 | 0.2 | 0.2 | 371.65 | 3396 |
| H7C238 | POGZ | Pogo transposable element with ZNF domain (Fragment) | 1 | 1 | 51.7 | 51.7 | 5.9725 | 60 |
| Q6ZMI0-4 | PPP1R21 | Isoform 4 of Protein phosphatase 1 regulatory subunit 21 | 1 | 1 | 2.2 | 2.2 | 42.132 | 363 |
| B1AN99 | PRSS3 | Trypsin-3 (Fragment) | 1 | 1 | 7.3 | 7.3 | 19.288 | 177 |
| Q9UL25 | RAB21 | Ras-related protein Rab-21 | 1 | 1 | 6.7 | 6.7 | 24.347 | 225 |
| Q5QPM0 | RALY | RNA-binding protein Raly (Fragment) | 1 | 1 | 5.3 | 5.3 | 18.66 | 171 |
| Q8IZ41-2 | RASEF | Isoform 2 of Ras and EF-hand domain-containing protein | 1 | 1 | 5.8 | 5.8 | 20.736 | 189 |
| Q9H2L5-3 | RASSF4 | Isoform 3 of Ras association domain-containing protein 4 | 1 | 1 | 4 | 4 | 28.689 | 251 |
| E9PNS2 | RBBP4 | Histone-binding protein RBBP4 | 1 | 1 | 22.6 | 22.6 | 7.5495 | 62 |
| A0A0U1RQH7 | RBM39 | RNA-binding protein 39 (Fragment) | 1 | 1 | 4.1 | 4.1 | 26.698 | 245 |
| D6R927 | RBPJ | Recombining-binding protein suppressor of hairless | 1 | 1 | 2.9 | 2.9 | 43.496 | 382 |
| F5H2L2 | RECQL | ATP-dependent DNA helicase Q1 (Fragment) | 1 | 1 | 9.2 | 9.2 | 14.634 | 131 |
| E7EVL6 | REPIN1 | Replication initiator 1 (Fragment) | 1 | 1 | 11 | 11 | 16.14 | 146 |
| P40938-2 | RFC3 | Isoform 2 of Replication factor C subunit 3 | 1 | 1 | 4.6 | 4.6 | 34.756 | 305 |
| H7C123 | RPL10 | 60S ribosomal protein L10 (Fragment) | 1 | 1 | 9.9 | 9.9 | 10.028 | 91 |
| E7EX53 | RPL15 | Ribosomal protein L15 (Fragment) | 1 | 1 | 6.8 | 6.8 | 15.722 | 133 |
| F8VYV2 | RPL18 | 60S ribosomal protein L18 | 1 | 1 | 8.3 | 8.3 | 15.639 | 133 |
| J3QR09 | RPL19 | Ribosomal protein L19 | 1 | 1 | 4.7 | 4.7 | 23.134 | 193 |
| K7EMA7 | RPL23A | 60S ribosomal protein L23a | 1 | 1 | 18.6 | 18.6 | 7.9232 | 70 |
| K7ERY7 | RPL27 | 60S ribosomal protein L27 | 1 | 1 | 20 | 20 | 4.3663 | 40 |
| P47914 | RPL29 | 60S ribosomal protein L29 | 1 | 1 | 9.4 | 9.4 | 17.752 | 159 |
| F2Z388 | RPL35 | 60S ribosomal protein L35 | 1 | 1 | 10.4 | 10.4 | 10.645 | 96 |
| H0Y5B4 | RPL36A | 60S ribosomal protein L36a | 1 | 1 | 8 | 8 | 13.228 | 112 |
| E9PP36 | RPL8 | 60S ribosomal protein L8 | 1 | 1 | 7.4 | 7.4 | 16.18 | 148 |
| P04844-2 | RPN2 | Isoform 2 of Dolichyl-diphosphooligosaccharide--protein glycosyltransferase subunit 2 | 1 | 1 | 2.3 | 2.3 | 67.723 | 615 |
| E5RIP1 | RPS20 | 40S ribosomal protein S20 | 1 | 1 | 23.9 | 23.9 | 5.4292 | 46 |
| P62701 | RPS4X | 40S ribosomal protein S4, X isoform | 1 | 1 | 3.4 | 3.4 | 29.597 | 263 |
| Q14684-2 | RRP1B | Isoform 2 of Ribosomal RNA processing protein 1 homolog B | 1 | 1 | 1.5 | 1.5 | 82.175 | 740 |
| P05109 | S100A8 | Protein S100-A8 | 1 | 1 | 11.8 | 11.8 | 10.834 | 93 |
| A3KN83-4 | SBNO1 | Isoform 4 of Protein strawberry notch homolog 1 | 1 | 1 | 1.6 | 1.6 | 80.851 | 732 |
| K4DIA1 | SCN5A | Sodium channel protein | 1 | 1 | 0.5 | 0.5 | 221.3 | 1962 |
| C9JZ65 | SERPINB4 | Serpin B4 (Fragment) | 1 | 1 | 5.7 | 5.7 | 24.373 | 211 |
| P23246-2 | SFPQ | Isoform Short of Splicing factor, proline- and glutamine-rich | 1 | 1 | 1.6 | 1.6 | 72.262 | 669 |
| J3QS74 | SKA2 | Family with sequence similarity 33, member A, isoform CRA_d | 1 | 1 | 23 | 23 | 7.1077 | 61 |
| F8VVM2 | SLC25A3 | Phosphate carrier protein, mitochondrial | 1 | 1 | 3.7 | 3.7 | 36.161 | 324 |
| Q9H2G2-2 | SLK | Isoform 2 of STE20-like serine/threonine-protein kinase | 1 | 1 | 0.8 | 0.8 | 138.99 | 1204 |
| J3KT85 | SMARCE1 | SWI/SNF-related matrix-associated actin-dependent regulator of chromatin subfamily E member 1 (Fragment) | 1 | 1 | 8.9 | 8.9 | 12.106 | 101 |
| G8JLG1 | SMC1A | Structural maintenance of chromosomes protein | 1 | 1 | 0.6 | 0.6 | 140.86 | 1211 |
| O75643 | SNRNP200 | U5 small nuclear ribonucleoprotein 200 kDa helicase | 1 | 1 | 0.5 | 0.5 | 244.5 | 2136 |
| M0R0G9 | SNRPA | U1 small nuclear ribonucleoprotein A | 1 | 1 | 11.5 | 11.5 | 10.083 | 87 |
| P08579 | SNRPB2 | U2 small nuclear ribonucleoprotein B'' | 1 | 1 | 4.9 | 4.9 | 25.486 | 225 |
| P35610-2 | SOAT1 | Isoform 2 of Sterol O-acyltransferase 1 | 1 | 1 | 1.8 | 1.8 | 58.13 | 492 |
| Q96RM1 | SPRR2F | Small proline-rich protein 2F | 1 | 1 | 12.5 | 12.5 | 7.8052 | 72 |
| Q8N5C6 | SRBD1 | S1 RNA-binding domain-containing protein 1 | 1 | 1 | 0.9 | 0.9 | 111.77 | 995 |
| O76094-2 | SRP72 | Isoform 2 of Signal recognition particle subunit SRP72 | 1 | 1 | 1.6 | 1.6 | 67.879 | 610 |
| P05455 | SSB | Lupus La protein | 1 | 1 | 2.7 | 2.7 | 46.836 | 408 |
| A0A087WWQ6 | SSX3 | Protein SSX3 | 1 | 1 | 4 | 4 | 22.846 | 202 |
| P53999 | SUB1 | Activated RNA polymerase II transcriptional coactivator p15 | 1 | 1 | 8.7 | 8.7 | 14.395 | 127 |
| E7ENN3 | SYNE1 | Nesprin-1 | 1 | 1 | 0.1 | 0.1 | 964.83 | 8392 |
| E7ENY2 | TANK | TRAF family member-associated NF-kappa-B activator (Fragment) | 1 | 1 | 8.9 | 8.9 | 21.915 | 190 |
| H7BYN3 | TFAM | Transcription factor A, mitochondrial (Fragment) | 1 | 1 | 4.6 | 4.6 | 25.567 | 219 |
| Q08188 | TGM3 | Protein-glutamine gamma-glutamyltransferase E | 1 | 1 | 1.3 | 1.3 | 76.631 | 693 |
| Q01085 | TIAL1 | Nucleolysin TIAR | 1 | 1 | 2.9 | 2.9 | 41.59 | 375 |
| Q13009-2 | TIAM1 | Isoform 2 of T-lymphoma invasion and metastasis-inducing protein 1 | 1 | 1 | 0.8 | 0.8 | 170.58 | 1531 |
| P0DN76 | U2AF1L5 | Splicing factor U2AF 35 kDa subunit-like protein | 1 | 1 | 5.4 | 5.4 | 27.872 | 240 |
| Q9BZM6 | ULBP1 | NKG2D ligand 1 | 1 | 1 | 4.9 | 4.9 | 27.996 | 244 |
| Q401N2-2 | ZACN | Isoform 2 of Zinc-activated ligand-gated ion channel | 1 | 1 | 2.6 | 2.6 | 29.616 | 266 |
| H3BNX3 |  | Uncharacterized protein (Fragment) | 1 | 1 | 3.1 | 3.1 | 31.769 | 289 |
